# Medical News and Items

**Published:** 1877-10

**Authors:** 


### MEDICAL NEWS AND ITEMS.

Reduced Prices of Medical Books.—*Lindsay and Blakiston*, publishers, announce that they have made very considerable reduction in the prices of many of their medical books to suit the times.

Dr. Vidal, physician at Jokoska, Japan, writes that, according to the formulæ of speech of the Japanese patients, a treasure would not suffice to pay the advice of so great a sage as the educated physician, wherefore the Japanese content themselves with not paying him for his services, but only allotting a few pence for medicine and the expenses of the visit; a sum so insignificant that more often the European physician would feel insulted by its being offered, and therefore contents himself with the profuse compliments of his clients. On the whole he does not consider a Japanese *clientèle* profitable; and, if cultivated at all, it could only be accepted as a supplement to a practice among the resident Europeans.

A Parisian pharmaceutist received the following homœopathic prescription to be dispensed:

| | |
|---|---|
| Lact. mammel. virgin, (milk from a virgin's breast) - - | 30 c. c. |
| Aq. stil. (aquæ distil.) - - | 150 c. c. |
| Alc. (alcohol) - - - | q́. s. |
| F. S. A. Dr. T. | |

We have just received a copy of the Eleventh Annual Announcement of the Chicago College of Pharmacy, and we are glad to congratulate the pharmacists on their evident indication of prosperity. The announcement itself is a decided improvement on its predecessors, and shows clearly that the executive committee are zealously working for the perfection of their particular branch of the department of medicine. One special feature in this year's announcement is the removal of the college to the North-west corner of Jackson and Wabash. In this central location the committee have secured a well lighted and well ventilated hall, which, instead of damping the courage of the most ardent student, as their other hall must have done, will contribute greatly towards rendering the lectures a pleasure and profit to all concerned. Attached to the lecture hall is abundant accommodation for laboratory, library and instrument rooms, and we have no doubt that the several committees appointed to superintend these departments will see that they are in keeping with the advance movement which the general committee has made.

We are of the opinion that many of our young men studying medicine would do well to take a course in pharmacy before commencing the study of medicine proper. It will not be denied that the more intimately we are acquainted with the agents we employ, the more skillfully are we able to adapt their various properties; and those properties can only become familiar to us by a prolonged and intimate association.

The college opens on the first of October. T.

Report on the Chicago Floating Hospital for the season 1877. By Wm. Rutter, M. D., physician to the hospital. The floating hospital which was announced to be opened upon the advent of hot weather, has, after a period of great usefulness, been discontinued for this season, having, as in previous seasons, proved a marked success in the purpose for which it was opened, viz.: To afford convalescent and otherwise sick women and children, an opportunity of enjoying the most needful and efficient restorative agent—"pure air," such being hardly accessible to the well-to-do in a large city like Chicago, and much less so to the poor and needy who are of necessity crowded together in close apartments, on narrow and poorly drained streets. As a rule, the patients who responded to the kind and urgent invitations extended from time to time to all by the directors, attest to the benefit derived from a sojourn on the ship. Thus, while in many instances proving a valuable aid to the efforts of the family physician, these "fresh air baths," as the visit to the hopital-ship may properly be termed, have undoubtedly hastened convalescence and counteracted the morbid influences extant in the city during the hot season. It may here be stated that this institution, whose privileges were free to all, was maintained solely by charitable contributions, and it is alone due to the general depression in trade that the period of its usefulness, to the regret of its hearty supporters and numerous friends, could not be extended over a greater length of time. But it will be seen by the figures below, that the attendance during its existence of six weeks was, nevertheless, quite large.

There attended during the season — 2,234 adults, 3,947 children—total, 6,190. Average for each day, 206⅓.

The thermometer ranged throughout the season on board the ship, as follows:

The indications taken down at 10, 12, 2 and 4 o'clock show the average for each day.

| | | | |
|---|---|---|---|
| July 16th | 78° | Aug. 2d | 76° |
| " 17th | 82° | " 3d | 75° |
| " 18th | 79° | " 6th | 77° |
| " 19th | 66° | " 7th | 80° |
| " 20th | 58° | " 8th | 79° |
| " 23rd | 76° | " 9th | 76° |
| " 24th | 81° | " 10th | 77° |
| " 25th | 84° | " 13th | 70° |
| " 26th | 76° | " 14th | 70° |
| " 27th | 80° | " 15th | 71° |
| " 30th | 77° | " 16th | 77° |
| " 31st | 80° | " 17th | 79° |
| Aug. 1st | 80° | | |

